# Childhood Predictors and Adult Life Success of Adolescent Delinquency Abstainers

**DOI:** 10.1007/s10802-015-0061-4

**Published:** 2015-08-13

**Authors:** N. Mercer, D. P. Farrington, M. M. Ttofi, L. Keijsers, S. Branje, W. Meeus

**Affiliations:** Research Centre Adolescent Development, Utrecht University, Heidelberglaan 1, 3584 CS Utrecht, The Netherlands; Cambridge Institute of Criminology, University of Cambridge, Cambridge, UK; Department of Developmental Psychology, Tilburg University, Tilburg, The Netherlands

**Keywords:** Abstainers, Delinquency, Adolescence, Developmental taxonomy, Social control, Life success

## Abstract

While much is known about adolescent delinquency, considerably less attention has been given to adolescent delinquency abstention. Understanding how or why some adolescents manage to abstain from delinquency during adolescence is informative for understanding and preventing adolescent (minor) delinquency. Using data from the Cambridge Study in Delinquent Development (*N* = 411 males) to compare abstainers, self-report delinquents and convicted delinquents we found five childhood factors (ages 8–10) that predicted adolescent abstention (ages 10–18). First, we find that adolescent abstainers possess characteristics opposite to those of convicted delinquents (namely, abstainers are high on honesty, conformity and family income). However, we also found that abstainers also share some childhood characteristics with convicted delinquents (namely, low popularity and low school achievement). A latent class analysis indicated that the mixed factors predicting abstention can be accounted for by two groups of abstainers: an adaptive group characterized by high honesty, and a maladaptive group characterized by low popularity and low school achievement. Further, validation of these two types of abstainers using data collected at age 48 suggested that adaptive abstainers outperform all other adolescents in general life success, whereas maladaptive abstainers only fare better than delinquent adolescents in terms of lower substance use and delinquency later in life.

Most adolescents engage in some form of delinquent or rule-breaking behavior (Moffitt et al. 2002). Indeed, decades of research have examined predictors of adolescent delinquent behaviour (for an overview, see: Farrington 1995; Loeber and Dishion 1983). Research has more recently focused on distinguishing between adolescents whose delinquent behavior persists well into adulthood and those who eventually desist (Loeber et al. 2007; Van Domburgh et al. 2009). As a result, much is known about the timing of (serious) delinquency onset, and eventual persistence or desistance. However, less attention has been paid to an alternative question: Can we predict which adolescents will manage to abstain from delinquency altogether?

A small group of adolescents, referred to as the abstainers (6–20 %), manage to avoid engaging in delinquent behaviors during adolescence (e.g., Brezina and Piquero 2007; Chen and Adams 2010; Johnson and Menard 2012; Moffitt et al. 2002). However, little is known about this unique group of adolescents and what may lead them to abstain from delinquent behavior. This limited knowledge is partly due to the relatively few studies on abstention, and even fewer studies on its early predictors. A better understanding of abstainers and how they manage to avoid delinquency during a period when it is most common is informative for adolescent delinquency prevention. Additionally, studying how or why some adolescents abstain is most informative when these abstainers are being compared to different types of delinquent adolescents. More specifically, we argue that instead of comparing abstainers and non-abstainers (delinquents), a further nuanced distinction within delinquent adolescents is needed to draw informative conclusions regarding not only abstention, but also the delinquency often considered adaptive in adolescents (Moffitt 2008). Therefore, when examining the potential childhood predictors of abstention, we consider a distinction between three groups of adolescents: adolescent abstainers, the majority of adolescents who engage in some delinquency, and the more serious adolescent offenders.

## Abstention Theory and Literature

### Predicting Abstention

We consider two alternative explanations for adolescent abstention based on childhood predictors: the linear hypothesis and the discrete group hypothesis. The linear hypothesis suggests that the factors that predict abstention will be the inverse of factors known to predict serious delinquents out of the majority of adolescents who engage in some delinquency. For example, if poor parent–child relationships predict delinquency, the most serious delinquents will have the poorest parent-child relationships and abstainers will have the strongest parent child relationships, with the majority of adolescents falling somewhere in between abstainers and serious delinquents. Alternatively, the discrete group hypothesis suggests that abstention is the result of unique factors unrelated to the distinction between different groups of delinquents. For example, abstainers may be shy adolescents, socially withdrawn, and excluded from their peer groups, whereas we have no such expectations for these characteristics to distinguish between who will be a serious delinquent compared to the majority of adolescents.

These two alternative explanations for the relationship between abstention and delinquency can be inferred from existing theory on adolescent delinquency. On the one hand, the linear hypothesis is derived from delinquency theories that provide implicit hypotheses for abstention based on their expectations of delinquency as a linear construct. An implication of viewing delinquency on a continuum is that abstention should be predicted by the same variables as offending, due to the presence of promotive factors (i.e., factors that lower the probability of offending, regardless of risk; Farrington et al. 2008). Social control theory (Hirschi 1969) is an example of a theory that implicitly supports the linear hypothesis. Specifically, social control theory hypothesizes that adolescents are equally likely to perceive delinquency as rewarding. However, adolescents vary in the strength of their social bonds and the costs of jeopardizing these bonds aid some adolescents in avoiding delinquency. Therefore, promotive factors, such as strong bonds to parents, school or conventional others, prevent adolescents from engaging in delinquency (abstainers), whereas delinquent adolescents have weak or poor bonds that do not aid in deterring them from delinquency. In fact most literature to date supports this framework, having found that abstainers generally have stronger bonds to parents (Johnson and Menard 2012), teachers (Piquero et al. 2005) or possess other promotive factors such as being high in moral beliefs (Brezina and Piquero 2007) or prosocial attitudes contrary to the poor parental relationships, poor school functioning, low moral beliefs and antisocial attitudes often related to delinquency.

On the other hand, theory directly pertaining to delinquency abstention during adolescence is limited: Moffitt’s developmental taxonomy is the only theory to date that has explicit hypotheses regarding abstainers and therefore forms the basis for the discrete group hypothesis in this paper. The developmental taxonomy (Moffitt 1993; Moffitt et al. 2002) expects that — motivated by their desire for autonomy — adolescents who engage in minor delinquency and rule breaking do so by mimicking (delinquent) peers. Following from this reasoning, the developmental taxonomy expects that some (minor) delinquency during adolescence is normative. Abstainers, therefore, are adolescents who have characteristics seen as undesirable by more popular peers, or that leave them reluctant, unable or restricted from joining (delinquent) peer groups leading to abstention from age-normative delinquency. There is some evidence in support of the discrete group hypothesis, as abstainers have been found to be fearful, shy, passive, unemotional, and have verbal communication difficulties in childhood (Owens and Slocum 2012). Furthermore, abstainers were found to be at least partially excluded from the popular (delinquent) peer groups during adolescence (Chen and Adams 2010; Rulison et al. 2014). Taken together, theory and literature point to two alternative explanations of abstention — the linear and the discrete group hypotheses — that will be examined in this study.

### Two Groups of Abstainers?

By examining the linear and discrete group hypotheses, we aim to provide insight into the relationship between abstention and delinquency to better understand the development of abstainers. Furthermore, we take this approach one step further, by suggesting that these two alternative hypotheses may actually address two different groups of abstainers. Indeed, the possibility of heterogeneity within abstainers has been discussed previously (see: Hendrix 2014; Johnson and Menard 2012). Furthermore, the implicit and explicit theories of abstention are divided on more than just the linear versus discrete group hypotheses about the factors that would predict abstaining. These two perspectives also differ on whether or not they would consider abstention to be adaptive behavior: the linear hypothesis may explain one group of adolescents who are characterized by promotive factors and adaptive functioning and the discrete group hypothesis may better represent a second group of abstainers characterized by unique factors and maladaptive functioning.

### Current Study

The main aim of the current study is to better understand adolescent delinquency abstainers by examining two alternative expectations for the childhood factors that predict abstention. Specifically, we will examine these plausible explanations for abstention when compared to different groups of adolescents who engage in different levels of delinquency with three exploratory hypotheses.

Our first hypothesis is the linear hypothesis: Abstainers will be predicted by the inverse of childhood factors that distinguish between the majority of adolescents and serious delinquents. Our second hypothesis is the discrete group hypothesis: Abstainers will be predicted by unique factors that do not distinguish between the majority of adolescents and serious delinquents. Finally, we consider a third hypothesis that the first two competing hypotheses may be reconciled by the existence of two different groups of abstainers.

Our study differs from previous research on early predictors of delinquency in that we have an overt focus on predicting abstaining compared to the majority of adolescents who engage in some delinquent behavior. Our paper also adds to the small body of abstention literature as one of the few papers to measure delinquency abstention across the entire span of adolescence (ages 10 to 18). Moreover, by combining literature and theory on adolescent delinquency abstention, this the first paper to test whether these two alternative expectations regarding the predictors and nature of abstainers may be reconciled by the existence of different groups of adolescent abstainers. Furthermore, we examine whether different groups of abstainers can be labeled as adaptive or maladaptive based on their achievement of developmental life tasks by age 48. Together these aspects of our study allow us to uniquely contribute to a better understanding of why aren’t all adolescents delinquent.

## Method

### Sample

The Cambridge Study in Delinquent Development (CSDD) is a prospective, multi-informant longitudinal survey of the development of offending and antisocial behavior in 411 males from South London. Data collection began in 1961–62 and the sample primarily consists of boys who were aged 8–9 and on the registers of six state primary schools within a one-mile radius of a research office that had been established.

Most of the boys (87 %) were Caucasian and have British origins. At age 8, 94 % of the boys could be described as working-class based on their father’s occupation (skilled, semi-skilled or unskilled manual workers), compared with the national figure of 78 % at that time. The majority of the boys were living in conventional two-parent families.

### Procedure

The boys were interviewed and given assessments at their schools at ages 8, 10 and 14. They were interviewed at the research office at 16, 18, and 21 and at home at 25, 32 and 48. The psychologists administering the assessments and the psychiatric social workers who conducted the interviews assured boys of the confidentiality of their assessments and answers. Attrition in the CSDD is negligible given the study’s duration: At age 14, 406 boys were assessed. At age 18, 389 of the original 411 were interviewed. Of the 22 missing, one had died, one could not be located, six were abroad, 10 refused and four more had parents who refused on their behalf; At age 48, 365 of the 394 still alive were interviewed (93 %).

The assessments in schools measured factors such as intelligence, personality and psychomotor impulsivity, whereas the interviews focused on topics such as living circumstances or leisure activities including drinking and drug use as well as self-reported delinquency. During the school assessments, peers were given questionnaires in which they rated the boys on characteristics such as popularity and daringness. Furthermore, teachers completed questionnaires when the boys were aged about 8, 10, 12 and 14. The teacher questionnaires addressed topics such as restlessness or poor concentration, school achievement and disruptive behavior in class.

In addition to the interviews and assessments with the boys, their peers and teachers, parents were also interviewed annually from when the boys were age 8 to approximately 15 years old. Although the mother was the primary informant, many of the boys’ fathers were also interviewed. These interviews conducted by psychiatric social workers provided information about subjects such as family income, family composition, parental employment histories and child-rearing practices (including discipline and supervision). Parents were also assured of the confidentially of their data. Finally, in order to obtain information regarding convictions for both the boys and their family members, searches were conducted in Criminal Record Office up to 1994 and in the Police National computer from then onwards.

The interviews at age 32 and 48 were approved by the Ethics Committee of the Institute of Psychiatry, Kings College London, and written informed consent was obtained from the participants. Earlier data collections were approved by the UK Home Office and verbal informed consent was obtained from the participants.

### Measures

#### Self-Report Delinquency

To measure self-report delinquency, offences were presented on cards, and the males were asked to sort the cards according to whether or not they had ever committed each act during a specified reference period, and if they had, at what age they had first and last committed these acts. From the age 14 interview (median age = 14.9) the following 6 items were used: burglary, theft of motor vehicles, theft from motor vehicles, theft from machines (e.g., slot machines), shoplifting and vandalism. During the age 18 interviews (median age = 18.7), the boys were asked to recall if they had engaged in any of the same 6 items in the last 3 years, if they had ever started fights in the last 3 years and if they had ever used drugs.^1^

#### Convicted Delinquency

Conviction offenses included in this study are the following 9 items which overlap with the self-reported delinquency: burglary, shoplifting, theft from motor vehicles, theft of motor vehicles, theft from machines, violent offenses, vandalism and drug offenses, as well as 10 other offenses: fraud, theft from work, other theft, robbery, suspected persons, weapons offenses, receiving offenses, serious motor vehicle offenses, threatening and sexual offenses recorded from age 10–18.

#### Group Identification

Based on our a priori criteria for abstention: a zero score on all included self-report and conviction items between ages 10–18, we were able to identify 49 boys who we could classify as adolescent abstainers. There were 239 boys who indicated they were involved in adolescent delinquency via self-report data, but who were not convicted by age 18. These boys were classified as the self-report delinquents and serve as the reference group for the remainder of the analyses. Finally, 117 boys were convicted for at least one offense between 10 and 18, and these boys were classified as convicted delinquents,^2^^.^^3^ The convicted delinquents had a mean variety score (*M* = 2.30, *SD* = 1.49) for convictions across adolescence. However, the convicted delinquents also reported significantly higher mean variety score (*M* = 5.35, *SD* = 2.83) for self-report acts compared to the self-report delinquents (*M* = 2.72, *SD* = 1.82), *t*(154.85) = 8.95, *p* < 0.001. The convicted delinquents also reported a greater frequency of self-report acts (*M* = 37.72, *SD* = 56.51) compared to self-report delinquents (*M* = 8.28, *SD* = 15.95), *t*(118.39) = 5.39, *p* < 0.001. Finally, the convicted delinquents also report a significantly higher number of violent acts (i.e., starting fights; *M* = 7.17, *SD* = 15.98) compared to the self-report delinquents (*M* = 1.45, *SD* = 6.19), *t*(127.21) = 3.66, *p* < 0.001. Together, these differences indicate that the convicted delinquents are indeed a group of more troubled, more seriously delinquent adolescents compared to the self-report delinquent group.

#### Childhood Predictors

Childhood predictors were included based on theory and previous literature on known risk factors for (serious) delinquency (e.g., Farrington and Ttofi 2011; Loeber et al. 2007; Piquero et al. 2010) as well as any additional predictors theory or literature may have suggested for abstention.

- *Individual Childhood Predictors*. Eighteen individual predictors were taken from parent, boy, teacher and peer interviews and questionnaires from ages 8–10. Therefore, we included: disobedience (*1 = overly-pliant to 3 = resistant to discipline*), combined peer ratings of popularity (*1 = least popular to 4 = most popular*), non-verbal IQ (*1 = 90 or below to 4 = 111 or above*), combined ratings of daring (*1 = least daring to 4 = most daring*), peer ratings of honesty (*1 = least honest to 4 = most honest*), psychomotor impulsivity (*1 = low to 5 = high*), concern with trying to be a credit to his parents (*1 = does not care to 3 = very concerned*), overall nervousness (*1 = low to 4 = high*), New Junior Maudsley Inventory (NJMI) extraversion (*1 = score 7 or less to 4 = score 12 or more*); NJMI neuroticism (*1 = 3 or less to 4 = 7 or more*), NJMI social conformity^4^ (*1 = 4 or less to 4 = 7 or more*), primary school achievement (*1 = low to 4 = high*) and lacking concentration (*1 = no to 2 = yes*).
- *Environmental Childhood Predictors.* Fourteen environmental predictors were extracted from parent and boy interviews. These factors are: parents interest in education (*1 = uninterested to 3 = very interested*); family income (*1 = inadequate to 3 = comfortable*); family size (*1 = no siblings to 6 = 5 or more siblings*); parental supervision (*1 = slack to 3 = rigid*); parents’ authoritarian attitude towards discipline (*1 = good to 4 = poor*); quality of parental marriage (*1 = bad to 3 = good*); overall mother nervousness (*1 = no symptoms to 3 = very nervous*); school delinquency (*1 = low to 3 = high*); criminal parent (*1 = no to 2 = yes*); delinquent older sibling (*1 = no to 2 = yes*).

#### Adult Outcomes

To measure adult outcomes for these adolescent groups, we used eight dichotomous indicators of life success measured at age 48 from The Life Success Score (Farrington et al. 2006). In this study, these indicators were separated into two domains and a cumulative domain score was calculated.

- *Self-report delinquency and substance use*. The first domain is representative of self-report delinquency and substance use in the past 5 years: not being involved in fights, low alcohol use (i.e., not driving under the influence, not a heavy nor a binge drinker), no drug use (not using cannabis or other drugs), and no self-reported delinquency (burglary, theft from vehicles, shoplifting, theft from machines and vandalism). Further, we use official conviction records from ages 19–48 to assess later life delinquency onset.
- *General life success*. The second domain is representative of general life success in the past 5 years: having satisfactory accommodation (i.e., whether he was a home owner, if the housing was of good quality and having moved less than three times in the last 5 years), having satisfactory employment (i.e., being currently employed, not of a low social class, had reasonable take home pay and no longer periods of unemployment in the last 5 years), having a satisfactory intimate relationship (i.e., living together or married for 5 years or more, not divorced in the last 5 years and generally getting on well with his partner) and satisfactory anxiety and depression scores (as measured by the General Health Questionnaire; Goldberg 1978). Satisfactory scores are scored with a 1 for each item leading to a satisfactory delinquency and substance use scale (0 = *min.* to 4 = *max.*) and a general life success scale (0 = *min.* to 4 = *max.*)

### Analytic Strategy

#### Linear Versus Discrete Hypotheses

In order to examine our linear and discrete group hypotheses for adolescent abstention, we need to be able to examine the variation in delinquency across the distribution of the childhood predictor variables (Farrington et al. 2008). For example, is the effect of parenting on delinquency equal across the different ranges of parenting quality? Or does poor parenting increase the probability of delinquency, whereas there is no distinction between good-enough parenting and excellent parenting in decreasing the probability of delinquency? This variation can be examined by testing the high and low ends of a variable separately using a risk and promotive factors approach. Previous literature defines a risk factor as any factor that increases the probability of offending (e.g., Loeber and Dishion 1983), whereas promotive factors are defined as those that lower the probability of offending, regardless of risk (Farrington et al. 2008; Loeber et al. 2007; Stouthamer-Loeber et al. 2002; Van der Laan et al. 2010). Based on the direction of effects found using either end of a variable, we can determine if each variable has independent risk, promotive or mixed effects. Further, by defining a variable as either risk or promotive empirically rather than a priori we are able to consider the possibility that certain variables can be risk factors for some adolescents and promotive factors for others ( Stouthamer-Loeber et al. 2002).

We followed the method proposed by Stouthamer-Loeber et al. (1993) by trichotomizing all non-dichotomous variables, aiming as closely as possible to represent the top 25 % (high) of the distribution and the bottom 25 % (low) of the distribution. The middle 50 % of the distribution is then the reference category. In doing so, we can empirically test the relationships between the dependent variable and different parts of the distribution of the independent variable. Trichotomization allows us to identify the possibility of non-linear relationships between predictors of abstention and delinquency in a straightforward, easy to interpret manner that is also not affected by any non-normality of variables (Van der Laan et al. 2010). Furthermore, Farrington and Loeber (2000) have shown that trichotomous categorization of variables has little effect on the overall conclusions about the importance of relevant variables. Two dichotomous environmental variables (i.e., criminal parent, delinquent sibling) and one individual factor were dichotomous (i.e., lacking concentration).

First, we examined the overall bivariate relationship between childhood predictors and adolescent group membership (abstainer, self-report delinquent, convicted delinquent) with Pearson’s Chi-Square (*X*^2^; trichotomized = 3 × 3, dichotomized = 2 × 3). Second, for variables that were generally related to group membership using an two-tailed exploratory alpha (*α* < 0.10),^5^ we further identified the nature of the effects (promotive, risk or mixed) by conducting logistic regressions separately for the high and low ends of the distribution (0 = *50 %*, 1 = *top or bottom 25 %*, *α* < 0.05). That is, we tested whether being in the high versus neutral, or low versus neutral, end of the variable predicted being an abstainer versus a self-report delinquent. We repeated this separately to test whether being in the high versus neutral or low versus neutral end of the variable predicted being a convicted delinquent versus a self-report delinquent.

#### Two Groups of Abstainers

Second, to test our final hypothesis, we conducted Latent Class Analyses (LCA; Nylund et al. 2007) in Mplus 7.1 to examine if there may be two groups of abstainers represented by the significant risk and promotive factors for abstention. Finally, we tested the validity of the LCA results by conducting ANOVAs to test mean differences on adult outcomes at age 48.

## Results

### Linear Versus Discrete Hypotheses for Abstainers Versus Self-Report Delinquents

To test the linear versus discrete hypotheses, we first examined if individual and environmental childhood factors could predict who would be an adolescent abstainer versus a self-report delinquent.

#### Individual Childhood Predictors

Out of the nine childhood predictors that were significant in the overall chi-square comparison, four of them distinguished between abstainers and self-report delinquents. All four of these effects were promotive (Table 1). More specifically, scoring in the highest 25 % of the sample on honesty (*b* = 0.83, *SE* = 0.38, *p* = 0.027) and conformity (*b* = 1.15, *SE* = 0.35, *p* = 0.001) and being in the lowest 25 % of the sample on primary school achievement (*b* = 1.10, *SE* = 0.41, *p* = 0.008) and peer ratings of popularity (*b* = 0.85, *SE* = 0.40, *p* = 0.035) all significantly increased the likelihood of being an adolescent abstainer compared to a self-report delinquent. There were no childhood factors in the individual domain that decreased the likelihood of being an abstainer compared to a self-report delinquent.

**Table 1:** Results of logistic regression models in which childhood predictors from the individual domain are predicting abstainers and convicted delinquents versus self-report delinquents

Predictor	Odds ratios	Effect	Odds ratios	Effect
	Abstainers		Convicted delinquents	
Popularity				
High	1.49	-	1.03	-
Low	2.34*	Promotive	1.76*	Risk
Non-verbal IQ				
High	1.33	-	0.82	-
Low	1.86	-	2.11**	Risk
Daring				
High	0.36	-	2.58***	Risk
Low	1.61	-	0.51*	Promotive
Honesty				
High	2.30*	Promotive	0.49*	Promotive
Low	0.65		2.12**	Risk
Concern with parents				
High	1.17	-	0.92	-
Low	0.54	-	3.23***	Risk
Neuroticism				
High	1.30	-	1.30	-
Low	1.20	-	0.52*	Promotive
Conformity				
High	3.16**	Promotive	0.84	-
Low	0.69	-	1.81*	Risk
School achievement				
High	1.85	-	0.52*	Promotive
Low	3.00**	Promotive	2.54**	Risk
Poor concentration				
Yes	0.96	-	1.72*	Risk

*Note.* ***p < 0.001 **p < 0.01 *p < 0.05, The Self-Report Delinquents are the reference group

#### Environmental Childhood Predictors

Only one environmental predictor out of the nine that were significant in the chi-square analyses was significantly able to distinguish between abstainers and the self-report delinquents in the binary logistic regression models. Scoring in the highest 25 % of the sample on family income (*b* = 0.73, *SE* = 0.35, *p* = 0.037) significantly increased the odds of abstaining, and therefore can be classified as a promotive effect.

### Linear Versus Discrete Hypotheses for Convicted Delinquents Versus Self-Report Delinquents

To test the linear versus discrete hypotheses, we also examined if individual and environmental childhood factors could predict who would be a convicted delinquent versus a self-report delinquent in adolescence.

#### Individual Childhood Predictors

All nine childhood predictors that were significant in the chi-square tests were able to significantly distinguish between convicted delinquents and self-report delinquents in the logistic regression models (Table 2). Only scoring in the lowest 25 % of neuroticism could be classified as a promotive factor (*b* = −0.66, *SE* = 0.32, *p* = 0.044). Three factors could be classified as mixed. Scoring in the highest 25 % on daring (*b* = 0.95, *SE* = 0.26, *p* < 0.001) and lowest 25 % on honesty (*b* = 0.75, *SE* = 0.28, *p* = 0.008) and school achievement (*b* = 0.93, *SE* = 0.28, *p* < 0.001) significantly increased the odds of convicted versus self-report delinquency. However, scoring in the lowest 25 % on daring (*b* = −0.67, *SE* = 0.34, *p = 0.*046) and highest 25 % on honesty (*b* = −0.72, *SE* = 0.35, *p* = 0.048) and school achievement (*b* = −0.66, *SE* = 0.32, *p* = 0.040) also decreased the odds of being convicted during adolescence. The final five predictors were classified as solely risk factors. More specifically, scoring in the lowest 25 % of the sample in popularity (*b* = 0.57, *SE* = 0.28, *p* = 0.042), non-verbal IQ (*b* = 0.75, *SE* = 0.27, *p = 0.*005), concern with being a credit to parents (*b* = 1.17, *SE* = 0.30, *p* < 0.001), conformity (*b* = 0.59, *SE* = 0.27, *p = 0.*027), and concentration (*b* = 0.54, *SE* = 0.24, *p = 0.*023) all lead to an increase in the odds of being an adolescent convicted delinquent versus self-reported delinquent.

**Table 2:** Results of logistic regression models in which childhood predictors from the environmental domain are predicting abstainers and convicted delinquents versus self-report delinquents

Predictor	Odds ratios	Effect	Odds ratios	Effect
	Abstainers		Convicted delinquents	
Education interest				
High	1.35	-	0.53*	Promotive
Low	0.89	-	1.51	-
Family income				
High	2.08*	Promotive	0.99	-
Low	1.48	-	2.52**	Risk
Family size				
High	0.63	-	2.15**	Risk
Low	1.29	-	0.78	-
Parental supervision				
High	1.74	-	0.68	-
Low	0.64	-	2.13**	Risk
Parent disharmony				
High	0.53	-	1.58	-
Low	0.80	-	0.53*	Promotive
School delinquency				
High	1.17	-	2.25**	Risk
Low	0.97	-	0.44**	Promotive
Temp. separation				
High	0.44	-	1.86	-
Low	0.63	-	0.76	-
Criminal parent				
Yes	1.84	-	4.68***	Risk
Delinquent sibling				
Yes	1.48	-	3.37***	Risk

*Note.* ***p < 0.001 **p < 0.01 *p < 0.05. The Self-Report Delinquents are the reference group

#### Environmental Childhood Predictors

Eight out of the nine childhood factors that were significant in the chi-square tests significantly predicted convicted delinquents versus self-report delinquents. There were one two purely promotive effects: being in the highest 25 % of the sample on parents’ interest in child’s education (*b* = −0.63, *SE* = 0.29, *p = 0.*029) and being in the lowest 25 % on parental disharmony (*b* = −0.65, *SE* = 0.31, *p = 0.*036) significantly decreased the odds of offending. There was one mixed effect: Being in the highest 25 % of school delinquency significantly increased the odds of being convicted in adolescence (*b* = 0.81, *SE* = 0.30, *p* = 0.007) whereas scoring in the lowest 25 % of school delinquency significantly decreased the odds of being convicted (*b* = −0.81, *SE* = 0.30, *p* = 0.006). On the other hand, there were five childhood factors that were designated as pure risk factors. Being in the lowest 25 % of the overall sample on family income (*b* =0.93, *SE* = 0.28, *p* = 0.001) and parental supervision (*b* = 0.76, *SE* = 0.29, *p* = 0.009), the highest 25 % of family size (*b* = 0.77, *SE* = 0.28, *p* = 0.006) having a criminal parent (*b* = 1.54, *SE* = 0.26, *p* < 0.001) and a delinquent sibling (*b* = 1.22, *SE* = 0.34, *p* < 0.001) all increased the odds of belonging to the convicted delinquent group.

Overall, comparing the five factors that predicted abstention to the 17 factors that predicted convicted delinquents from self-report delinquents we found three factors that support our linear hypothesis. Specifically, high scores on honesty, conformity and income separate abstainers and from the self-report delinquents; low scores on honesty, conformity and income also separate convicted delinquents from the self-report delinquents. The additional two factors that predict abstainers from self-report delinquents, low popularity and low school achievement do not fully support the discrete group hypothesis, as they are not unique factors. However, they do indicate the possibility of non-linear relationships in predicting abstention.

### Two Groups of Abstainers

In order to test the hypothesis that abstention may be best represented by two different groups of abstainers, latent class analyses were conducted on the five childhood promotive factors that predicted abstention. Following previously specified criteria for class selection (see: Nylund et al. 2007) a two-class solution was a better fit to the data than a one-class solution. The two-class solution had a lower sample size adjusted Bayesian Information Criterion (BIC; 2 class SSA-BIC = 296.364, 1 class SSA-BIC = 309.812) and significant Vuong-Lo-Mendell-Rubin Likelihood Ratio Test (*p* = 0.036). Furthermore, the two-class solution had excellent entropy (0.94) indicating a clear distinction between the two classes.

Table 3 indicates the number of boys in each class who are in the top 25 % of the sample on honesty, conformity and family income and in the bottom 25 % of the sample school achievement and popularity. The first class (*n* = 27) is characterized by more boys with adaptive factors, namely scoring in the highest 25 % on honesty, which would be expected by theories suggesting that adolescent abstention is an adaptive alternative to delinquency. The second class (*n* = 22) is represented by all of the abstaining boys who score in the bottom 25 % of low popularity, as well as the majority who do poorly in school. This group is consistent with the idea that adolescent abstention may be a reflection of maladaptive tendencies.

**Table 3:** The number of boys in each abstainer class in the top 25 % of significant childhood promotive factors that predicted abstention from self-report delinquency

Childhood predictor (top 25 %)	Abstainer class 1 (*n* = 27)	Abstainer class 2 (*n* = 22)
High honesty (Pe)	16	4**
High conformity (C)	15	7
High family income (Pa)	15	7
Low popularity (Pe)	0	19***
Low school achievement (T)	4	10*

*Pe* peer report, *C* child report, *Pa* parental report, *T* teacher/school report

*Note:* ***p < 0.001 **p < 0.01,*p < 0.05 *X* ^2^ contingency tables test for significance

### Adult Outcomes

**Fig. 1:**
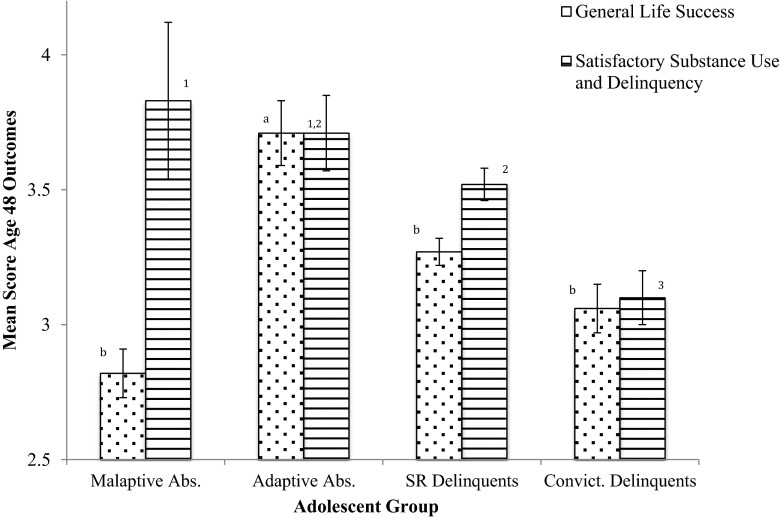
Pro-rated means and standard errors for cumulative scores of general life success and substance use and delinquency at age 48 for each adolescent trajectory. Means sharing the same superscript are not significantly different from each other at *p* < 0.05

As the first study to empirically indicate the possibility of two different types of adolescent abstainer groups based on competing linear and discrete group hypotheses, we tested the validity of this classification by examining potential differences in later life outcomes. A one-way ANOVA concluded that there were significant overall between group differences for general life success, Welch’s *F*(3, 50.03) = 5.46, *p* = 0.003, as well as for self-reported substance use and delinquency Welch’s *F*(3, 56.42) = 11.32, *p* < 0.001 at age 48. Figure 1 shows the pro-rated mean and standard errors for general life success and satisfactory substance use and delinquency. Games-Howell post-hoc tests show that adaptive abstainers were significantly better off in general life success represented by satisfactory employment, cohabitation and well-being in terms of anxiety and depression at age 48 compared to the maladaptive abstainers. Furthermore, the adaptive abstainers also report significantly better life success compared to self-report delinquent and convicted adolescents. Mean level differences between the maladaptive abstainers and the delinquency groups are non-significant.

In terms of low substance use and delinquency at age 48 the two abstainer groups do not significantly differ. However, the maladaptive abstainers reported significantly more satisfactory scores in this domain compared to both delinquents and convicted delinquents, whereas the adaptive abstainers only reported a significantly better mean compared to the convicted adolescents (Fig. 1).

Furthermore, we calculated the total frequency of convictions in adulthood based on official records from age 19 to age 48. A one-way ANOVA indicated there were overall significant between group differences Welch’s *F* (3, 69.36) = 17.08, *p* < 0.001. Games-Howell post-hoc tests showed that the convicted adolescents had a significantly higher mean number of adult convictions (*M* = 3.38, *SD* = 4.57) than all other adolescents. The maladaptive abstainers (*M* = 0.48, *SD* = 1.25), adaptive abstainers (*M* = 0.15, *SD* = 0.46) and self-report delinquents (*M* = 0.43, *SD* = 1.07) did not significantly differ from each other. Results were identical when considering incidence of conviction rather than frequency. A one-way ANOVA indicated there were overall significant between group differences, Welch’s *F* (3, 60.71) = 24.64, *p* < 0.001. Convicted adolescents had a higher incidence of adult convictions (*n* = 68) than all other groups. Again, the maladaptive abstainers (*n* = 4), adaptive abstainers (*n* = 3) and self-report delinquents (*n* = 44) did not significantly differ from each other in incidence of adult convictions.

## Discussion

The primary aim of this paper was to determine which childhood factors could predict adolescent abstainers compared to adolescents who engage in self-report delinquency. More specifically, we used a risk and promotive factors trichotomization approach to test two plausible alternatives leading to abstention: We found support for the linear hypothesis — three predictors of abstention were the promotive ends of risk factors that also predict delinquency. However, we only found partial support for the discrete group hypothesis. We did not find that abstainers possessed unique childhood factors. Instead, we found that the remaining two characteristics of abstainers are shared with convicted delinquents. This finding still supports the discrete group hypothesis idea of a non-linear relationship between abstention and delinquency, although not predicted by unique factors. In addition, we found that there are two different groups of abstainers: an adaptive and a maladaptive group. Furthermore, in validating these two distinct abstainer groups, we found that they differed in their achievement of normative developmental life tasks. For instance, adaptive abstainers outperformed the maladaptive abstainers in general life success at age 48, a domain representing having satisfactory accommodation, employment, intimate relationships and positive mental health, but they did not differ in their use of alcohol, drugs, self-report delinquency or involvement in fights.

### Linear Versus Discrete Hypotheses

In terms of the support found for the linear hypotheses that childhood predictors of abstention are the inverse of predictors of delinquency, three of the five promotive factors that predict abstention compared to delinquency (high conformity, high honesty, high family income) are the promotive end of factors in which the bottom 25 % were risk factors for convicted delinquency (low conformity, low honesty, low family income). These findings are consistent with previous research which found that abstainers tend to have high moral beliefs (Brezina and Piquero 2007) or that delinquents tend to have delayed moral judgment development compared to non-delinquents (Stams et al. 2006) as well as research that generally suggests abstainers possess positive, protective factors when compared to delinquent adolescents (e.g., Brezina and Piquero 2007; Chen and Adams 2010; Piquero et al. 2005).

Furthermore, contrary to the expectations of the discrete group hypothesis: the remaining two childhood predictors of abstention were not unique but were identical to two predictors of convicted adolescents. Indeed, one benefit of the trichotomization approach is that it can identify factors that are risk factors for one group and promotive factors for another ( Stouthamer-Loeber et al. 2002). Although we did not anticipate that low popularity and low school achievement would predict adolescent abstainers and convicted adolescents, these findings are in line with previous research suggesting that abstainers may be socially isolated (Owens and Slocum 2012; Shedler and Block 1990) or have fewer friends (Chen and Adams 2010) when compared to non-abstaining adolescents. Additionally, some previous research has also suggested that abstainers may possess neurocognitive impairments (Owens and Slocum 2012), which may be consistent with our finding that (some) abstainers have lower school achievement than self-report delinquents. Overall, it seems abstention is primarily predicted by individual characteristics of the child.

### Two Groups of Abstainers

Our final hypothesis suggested that competing expectations for the factors that would lead to abstention could be explained by the existence of two different types of abstainers. Although we did not expect that abstainers and convicted offenders would be predicted from the self-report delinquents by their low scores on popularity and school achievement, this finding points to the presence of non-linear predictors of abstention and delinquency and suggests that there are two different types of abstainers. Indeed, we did observe two groups of abstainers in the data: one high on honesty — the adaptive abstainers; and the other unpopular and doing poorly at school — the maladaptive abstainers. We speculate that the mechanisms leading to abstention may be different between the two groups. For instance, the adaptive abstainers may be simply unwilling to engage in these normative delinquent behaviors with a host of individual resources available to support them (see also: Brezina and Piquero 2007; Cook et al. 2009), whereas the maladaptive abstainers may be excluded from or unable to join their peers in these activities due to social, cognitive or other impairments (see also: Owens and Slocum 2012; Shedler and Block 1990).

### Adult Outcomes

Because we were the first to model two different groups of abstainers based on these competing theoretical hypotheses, we wanted to validate the distinction between these two groups using information on later life outcomes collected at age 48. Notwithstanding the fact the group sizes were small, when it comes to overall life success, the adaptive abstainers do better than all other adolescent groups (including the maladaptive abstainers). We suggest that this composite score of success in employment, accommodation, intimate relationships and mental health represents the healthy achievement of the main developmental tasks in life. On the other hand, adult outcomes measures at age 48 indicate that the maladaptive abstainers, despite the childhood predictors they share with convicted delinquents, outperform both delinquent and convicted adolescents (but not the adaptive abstainers) on measures of satisfactory self-report delinquency and substance use later in life. These mean differences seem to suggest that despite shared childhood predictors with convicted delinquents, it is unlikely that the maladaptive abstainers become more violent or develop addictions later in life compared to the two groups of adolescent delinquents.

However, previous research in the CSDD has found that a small group of adult-onset offenders do exist, and that these men are characterized by withdrawn or nervous behavior that acts as a buffer against delinquency in adolescence but wears off later in life (Zara and Farrington 2010). Furthermore, adult-onset offenders are more likely to have a lower IQ and poor school achievement compared to non-offenders (Zara and Farrington 2009). Given the similarities in profile descriptions, it is important to consider whether the maladaptive abstainers are likely to become adult-onset convicted offenders. Although results indeed showed that some men were convicted for the first time after age 18, the vast majority (but not all) of these men belong to the self-report delinquency group. Importantly, these findings indicate that adult-onset hypotheses do not entirely account for either abstainer group irrespective of the similarities that adult-onset offenders in previous research may share with our group of maladaptive abstainers.

Overall, our findings suggest that consistent with the childhood factors that differentiated these two groups: the adaptive abstainers appear to be the success stories when it comes to later life development, whereas the maladaptive abstainers may be socially or cognitively impaired leading to failure in their achievement in relationship, vocational, mental health and/or financial domains when compared to the adaptive abstainers. Although there were no unique predictors of abstention, the finding that two subgroups of abstainers exist, one discrete and maladaptive and one linear and adaptive partially supports the developmental taxonomy’s expectations for a discrete group of maladaptive abstainers. Taken together, the results of this study question the validity of an often-used generalization from single behavioral outcomes (i.e., delinquent versus non-delinquent) to general development (i.e., unhealthy versus healthy). Instead we should examine how similar or different these adolescents, their characteristics, and environments are leading to their divergent pathways into an adulthood characterized by relatively lower life success.

### Limitations

Although this study is the first to examine risk and promotive factors predicting abstention versus different levels of delinquency, using multi-informant prospective longitudinal data spanning across the life course from age 8 to age 48, the findings from our study should be interpreted alongside the following limitations: The CSDD is a prospective study of a relatively homogeneous working class group of mostly white South London males from two-parent family homes and therefore, is unlikely to have comparable variation in constructs such as SES, family structure or ethnicity to be fully generalizable to other contexts or more representative population samples and gives us no indication of predictors of abstention in adolescent females. Additionally, self-report delinquency was only measured at two time points during adolescence (age 14 and 18), which precluded conducting longitudinal group based trajectory analyses to define our different delinquent groups. Additionally, this paper does not address childhood-limited offending. Although this was outside the theoretical scope of this paper, it is an interesting avenue for future research to consider. The CSDD was designed when much was known about the risk factors of offending, and therefore includes a wealth of childhood factors related to risk for delinquency. At the time, little was known about potential promotive or unique factors predicting abstention and therefore the variable list is less comprehensive in these aspects. Moreover, to further strengthen our finding that individual characteristics may be more important than environmental factors in predicting abstention more detailed measures of the peer context should be tested. Indeed, research suggests the peer context, and in particular peer delinquency is an important environmental context for the prediction of (minor) delinquency (Farrington 2003; Warr and Stafford 1991). Finally, this study took an exploratory approach to examining this question by considering plausible competing hypotheses using a number of possible childhood predictors. Therefore, future research should focus on the subset of predictors we have identified to provide confirmatory results. Indeed, whether these findings, and in particular the size of the two different groups of abstainers and their different childhood predictors and adult outcomes, would be observed in different samples using different measures is a question which should be addressed by future research.

## Conclusion

By focusing on abstention, this paper represents a paradigm shift in the delinquency literature that is meant to shed light on a question that is often taken for granted — why aren’t all adolescents delinquent? Overall, we found that abstention is primarily predicted by individual characteristics of the child, whereas being convicted by age 18 is predicted by both individual and environmental risk factors. Consistent with the linear hypothesis, whereas high honesty, high conformity, and high family income predicted abstention, low honesty, low conformity, and low family income predicted convicted delinquency. However, somewhat inconsistent with expectations derived from group-based theory, we did not identify any unique predictors of adolescent abstention. Instead, we found that abstainers and convicted offenders were both less popular and had poor school achievement when compared to self-report delinquents. Further, it appears that competing theories about the nature of abstainers can be reconciled by the observation of two abstainer groups: first, adaptive abstainers, those who can be predicted by the inverse of delinquency and who are the most successful group in later adulthood. Second, the maladaptive abstainer, a clinically relevant and discrete group who share childhood predictors with convicted adolescents, who do not become delinquent but instead may possess social or cognitive impairments or otherwise problematic psychosocial profiles that negatively affect their relational, vocational, and general well-being well into adulthood. This group of adolescents and the process by which they are identified markedly points to the necessity to consider the implications of problem behavior (or lack thereof) within a larger developmental framework. Overall, results suggest that abstainers can be either conformists who possess protective factors or outcasts who may be excluded from the delinquent behavior they may have otherwise participated in. Further research should take care to examine the possibility of shared beginnings in abstention and convicted offending for a small group of maladaptive abstainers.

## Footnotes

1 Two additional offenses were measured but excluded from this study: assault at age 14 (because the level nor direction of involvement (victimization) were not clear) and fraud at age 14 because it primarily consisted of not paying the (full) fare on public transportation which is substantially more minor than other self-report items which could all theoretically result in criminal convictions.

2 The age when the offense was committed, and not the actual conviction date was recorded and used to determine adolescent convictions.

3 6 males were excluded because they did not have any self-report data or complete conviction data. The current groups include males with at least one measurement point (age 14 or 18) of self-report delinquency data and/or complete conviction data (ages 10–18).

4 Originally titled the NMJI Lie scale, previous literature suggests lie measures in children better represent acquiescent or social conformist behavior, and we have retitled it accordingly (see: Jones and Francis 1995 for further discussion). Further, this measure is correlated with honesty (*r* = 0.19, *p* < 0.001) and daring (*r* = −0.13, *p* = 0.01) in the directions expected by measures of conformity rather than lying.

5 The following variables were not included for further analysis based on this exploratory criterion: mother nervousness, boy nervousness, extraversion, disobedience, psychomotor impulsivity and authoritarian parents.

## References

[CR1] Brezina T, Piquero AR (2007). Moral beliefs, isolation from peers, and abstention from delinquency. Deviant Behavior.

[CR2] Chen X, Adams M (2010). Are teen delinquency abstainers social introverts?: A test of Moffitt’s theory. Journal of Research in Crime and Delinquency.

[CR3] Cook EC, Buehler C, Henson R (2009). Parents and peers as social influences to deter antisocial behavior. Journal of Youth and Adolescence.

[CR4] Farrington DP (1995). The development of offending and antisocial behavior from childhood: key findings from the Cambridge study in delinquent development. Journal of Child Psychology and Psychiatry.

[CR5] Farrington DP (2003). Developmental and life-course criminology: key theoretical and empirical issues – the 2002 Sutherland award address. Criminology.

[CR6] Farrington DP, Loeber R (2000). Some benefits of dichotomization in psychiatric and criminological research. Criminal Behavior and Mental Health.

[CR7] Farrington, D. P., & Ttofi, M. M. (2011). Protective and promotive factors in the development of offending. In T. Bliesener, A. Beelmann, & M. Stemmler (Eds.), *Antisocial behavior and crime: Contributions of developmental and evaluation research to prevention and intervention* (pp. 71–88). Cambridge, Mass: Hogrefe.

[CR8] Farrington DP, Coid JW, Harnett L, Jolliffe D, Soteriou N, Turner R, West DJ (2006). Criminal careers up to age 50 and life success up to age 48: new findings from the Cambridge study in delinquent development.

[CR9] Farrington DP, Loeber R, Jolliffe D, Pardini D, Loeber R, Farrington DP, Stouthamer-Loeber M, Raskin White H (2008). Promotive and risk processes at different life stages. Violence and serious theft: Development and prediction from childhood to adulthood.

[CR10] Goldberg D (1978). Manual of the general health questionnaire.

[CR11] Hendrix, J. A. (2014). *Angels and loners: An examination of abstention processes and abstainer heterogeneity* (Doctoral Dissertation). Retrieved from North Carolina State University Dissertations Database. (1840.16/9891)

[CR12] Hirschi T (1969). Causes of delinquency.

[CR13] Johnson MC, Menard S (2012). A longitudinal study of delinquency abstention differences between life-course abstainers and offenders from adolescence into adulthood. Youth Violence and Juvenile Justice.

[CR14] Jones SH, Francis LJ (1995). The relationship between Eysenck’s personality factors and attitude towards truancy among 13–15 year olds in England and Wales. Personality and Individual Differences.

[CR15] Loeber R, Dishion T (1983). Early predictors of male delinquency: a review. Psychological Bulletin.

[CR16] Loeber R, Pardini DA, Stouthamer-Loeber M, Raine A (2007). Do cognitive, physiological, and psychosocial risk and promotive factors predict desistance from delinquency in males?. Development and Psychopathology.

[CR17] Moffitt TE (1993). Adolescence-limited and life-course-persistent antisocial behavior: a developmental taxonomy. Psychological Review.

[CR18] Moffitt TE, Cullen FT, Wright JP, Blevins KR (2008). A review of research on the taxonomy of life-course persistent versus adolescence-limited antisocial behavior. Taking stock, the status of criminological theory.

[CR19] Moffitt TE, Caspi A, Harrington H, Milne BJ (2002). Males on the life-course-persistent and adolescence-limited antisocial pathways: follow-up at age 26 years. Development and Psychopathology.

[CR20] Nylund KL, Asparouhov T, Muthén BO (2007). Deciding on the number of classes in latent class analysis and growth mixture modeling: a Monte Carlo simulation study. Structural Equation Modeling.

[CR21] Owens JG, Slocum LA (2012). Abstainers in adolescence and adulthood: exploring the correlates of abstention using Moffitt’s developmental taxonomy. Crime & Delinquency.

[CR22] Piquero AR, Brezina T, Turner MG (2005). Testing Moffitt’s account of delinquency abstention. Journal of Research in Crime and Delinquency.

[CR23] Piquero AR, Farrington DP, Nagin DS, Moffitt TE (2010). Trajectories of offending and their relation to life failure in late middle age: findings from the Cambridge Study in Delinquent Development. Journal of Research in Crime and Delinquency.

[CR24] Rulison KL, Kreager DA, Osgood DW (2014). Delinquency and peer acceptance in adolescence: a within-person test of Moffitt’s hypotheses. Developmental Psychology.

[CR25] Shedler J, Block J (1990). Adolescent drug use and psychological health: a longitudinal inquiry. American Psychologist.

[CR26] Stams GJ, Brugman D, Deković M, van Rosmalen L, van der Laan P, Gibbs JC (2006). The moral judgment of juvenile delinquents: a meta-analysis. Journal of Abnormal Child Psychology.

[CR27] Stouthamer-Loeber M, Loeber R, Farrington DP, Zhang Q, Van Kammen W, Maguin E (1993). The double edge of protective and risk factors for delinquency: interrelations and developmental patterns. Development and Psychopathology.

[CR28] Stouthamer-Loeber M, Loeber R, Wei E, Farrington DP, Wikström POH (2002). Risk and promotive effects in the explanation of persistent serious delinquency in boys. Journal of Consulting and Clinical Psychology.

[CR29] Van der Laan AM, Veenstra R, Bogaerts S, Verhulst FC, Ormel J (2010). Serious, minor, and non-delinquents in early adolescence: the impact of cumulative risk and promotive factors. The TRAILS study. Journal of Abnormal Child Psychology.

[CR30] Van Domburgh L, Loeber R, Bezemer D, Stallings R, Stouthamer-Loeber M (2009). Childhood predictors of desistance and level of persistence in offending in early onset offenders. Journal of Abnormal Child Psychology.

[CR31] Warr M, Stafford M (1991). The influence of delinquent peers: what they think or what they do?. Criminology.

[CR32] Zara G, Farrington DP (2009). Childhood and adolescent predictors of late onset criminal careers. Journal of Youth and Adolescence.

[CR33] Zara G, Farrington DP (2010). A longitudinal analysis of early risk factors for adult‐onset offending: what predicts a delayed criminal career?. Criminal Behavior and Mental Health.

